# Trophic status determination of the Egyptian Eastern Mediterranean Sea based on phytoplankton diversity and their biochemical contents

**DOI:** 10.1007/s10661-023-11690-z

**Published:** 2023-08-16

**Authors:** Mona M. Ismail, Mohamed H. Diab, Mostafa M. El-Sheekh

**Affiliations:** 1 National Institute of Oceanography and Fisheries, NIOF, Cairo, Egypt; 2 Botany Department, Faculty of Science, Tanta University, Tanta, Egypt

**Keywords:** Biodiversity, Bioindicator, Eastern harbour, Macromolecules, Pollution indices

## Abstract

The present study attempted to test the applicability of using phytoplankton as a bioindicator for assessing water quality along the Eastern Alexandria coast, Egypt. Eight stations were selected to cover the different characteristics of seawater during the summer 2022. Six algal groups were detected in different ratios: Bacillariophyceae, Cyanophyceae, Dinophyceae, Chlorophyceae, Silicoflagellata, and Euglenophyceae. Phytoplankton abundance was found highest at the Port Said (PS) beach, and the lowest abundance was detected at Ras El Bar (RB) beach. According to the evaluation of phytoplankton abundance, all the studied stations are oligotrophic state except PS was eutrophic. Based on Chl *a* concentration, the selected stations could be described as good-bad from west to east. The Shannon diversity and Pielou evenness indexes classified the studied stations as being moderate pollution except for PS and RB stations. The existence of organic pollution indicators species like *Navicula*,* Synedra*, and *Euglena* is a warning indication of the declining water quality especially in B and M stations. The biochemical compositions of the collected phytoplankton were spatial fluctuated. Protein and carbohydrates were the dominant macromolecules in the phytoplankton community compared with lipids. These macromolecules are used to assess trophic states whereas the ratio between protein and carbohydrate > 1 represents a healthy coast as observed in some stations. Thus, phytoplankton should be considered a bioindicator within Water Framework Directive monitoring programs for the Mediterranean Sea. For the long-term monitoring of the Mediterranean Sea’s ecosystem, it is recommended that the macromolecules of phytoplankton should be determined.

**Supplementary Information:**

The online version contains supplementary material available at 10.1007/s10661-023-11690-z.

## Introduction

The Mediterranean Sea is a well-known oligotrophic body of water that is deficient in nutrients and abundant in dissolved oxygen. The oligotrophic state rises from the west to the east. The pattern may have changed during the past several years due to changes in water properties, may be as a result of human activity (Christaki et al., 2011). Urbanization and tourism cause sewage pollution along the shore, raising seawater temperatures and salinities in both the eastern and western basins (Bethoux & Gentili, 1999). Notably, the United Nations Environment Programme (UNEP) reported that 650 million tonnes of sewage are discharged annually into the Mediterranean Sea (Karadirek et al., 2019). In addition to the persistent construction activities, rapid industrial development, and the continuous surge in population along Meditranean coast, Egypt, the seawater in this region has become increasingly polluted, making it one of the most contaminated areas in the Eastern Mediterranean Sea (Heger et al., 2022). One of the most significant regions along the Egyptian Mediterranean coast is the Alexandria coastal zone, which has a number of beaches. It extended from El-Dekhaila in the west to Abu Qir in the east (Dango et al., 2015). On the side, Ras El-Bar is located at the Mediterranean Sea’s Damietta Branch’s mouth, where fresh water from the Nile mixes with salt water from the Mediterranean. The Damietta estuary receives discharges from sewage treatment facilities like the Ras El-Bar Sewage Plant, agricultural runoff, industrial operations like the Moboco Fertiliser Plant, and heavy boat traffic at Ezbt Elborg (Abdel Galil et al., 2020). While Baltim Beach, one of the most significant public beaches fronting the center portion of the Nile Delta, is situated on Egypt’s northern shore, 11.5 km east of the Burullus lagoon inlet. Also, the Port Said coast experiences the influence of two significant water bodies, namely the Suez Canal and Lake Manzalah, resulting in predominantly Mediterranean-origin surface water. Extensive literature indicates that a substantial number of erythrean species have migrated through the Suez Canal, invading the Eastern Mediterranean (Madkour, 2000). The degree of freshwater influx caused by the outflowing lake water exhibits seasonal variation depending on the prevailing wind patterns in the area, predominantly from the north to northwest, with velocities ranging between 6 and 14.5 knots (Amer, 1999).

Recently, there seems to be interest in the Mediterranean Sea’s diverse phytoplankton, which is changing rapidly as a result of pollution, ship traffic, climate change, introduced species, and shifts in the native species distribution (El-Dahhar et al., 2021; Ismail et al., 2022).

The phytoplankton communities play an important role in marine biodiversity and productivity, as well as in monitoring environmental changes. Whereas, their abundance and structure are changed in response to environmental parameters so they can be utilized as a bioindicator of water quality and the degree of eutrophication (Kim et al., 2020). Since both biotic and abiotic environmental conditions have a significant impact on the succession and abundance of phytoplankton in maritime environments, these changes play an enormous role in coupling multiple nutrient cycles in marine ecosystems. Whereas, phytoplankton assemblage fluctuation is explained by many factors like nutrients supply, light variation, and mixing conditions (Litchman et al., 2015). As a result, the phytoplankton abundance, species composition, richness, evenness, and geographical and temporal dispersion are a reflection of the biological integrity or environmental well-being of a given body of water (Effiong et al., 2018). Also, some indicator algal species serve as crucial indicators of water pollution (Atıcı & Akiska, 2005).

Moreover, phytoplanktons are the main primary producer of carbon in marine ecosystems, and subsequently generate many essential biomolecules like total carbohydrates, proteins, and lipids within the phytoplankton biomass, which are potentially highly bioactive (Heraud et al., 2008; Ismail et al., 2022; Kim et al., 2022), and can be a useful predictor of their nutritional value (Ahn et al., 2019; Ismail et al., 2022).The changes in the main biochemical composition are mostly controlled by growth phase, species composition, and environmental parameters (Ahn et al., 2019; Ismail et al., 2022). The food quality for higher trophic levels is significantly impacted by any change in the biochemical makeup of the phytoplankton (Kim et al., 2022).

Several local studies have concentrated on phytoplankton community structure in one or more areas along the Eastern Alexandria coast (El-Sayed et al., 2019; El-Serehy et al., 2014; Gharib & Dorgham, 2000; Hussein, 2008; Ismail & Ibrahim, 2017; Labib et al., 2023).

This study looks into the spatial variation of phytoplankton community composition and their macromolecules composition (carbohydrate, protein, and lipid) in relation to the physico-chemical parameters of the Eastern Egyptian Mediterranean coast, from the west (Eastern Harbour) to the east (Port Said). This study also provides insights into the difference in water quality by using multi-statistical approaches and indices that could be helpful for management studies of this Mediterranean coastal environment.

## Materials and methods

### Sampling site

**Fig. 1:**
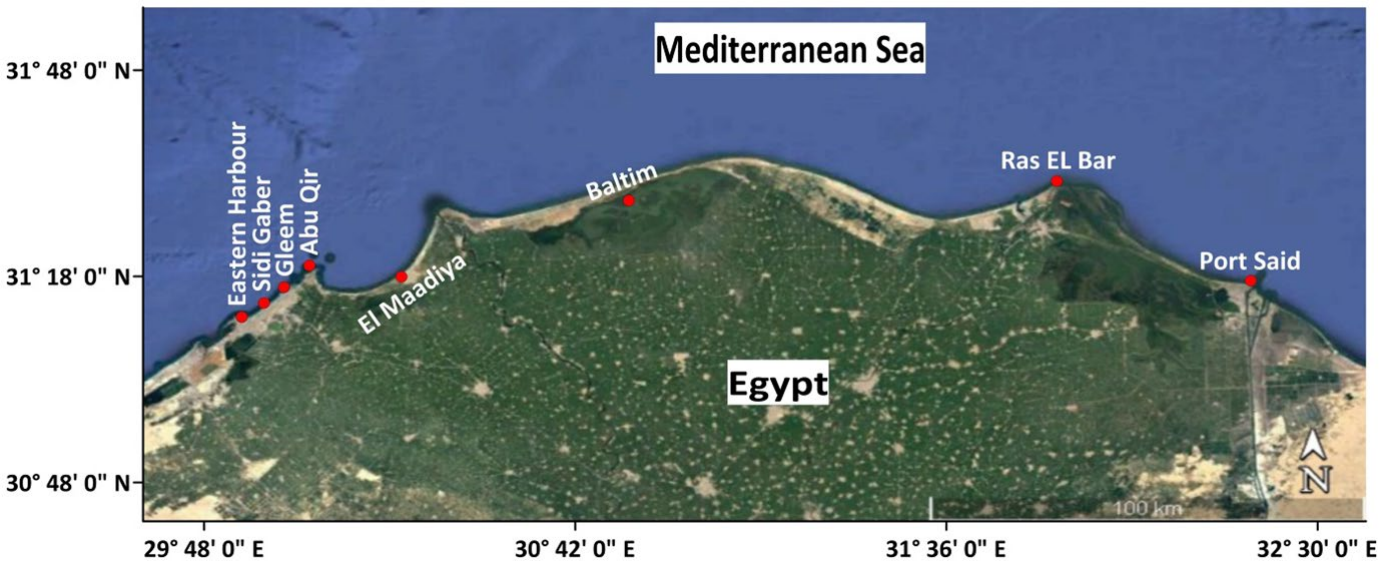
Map of the selected stations

Water and phytoplankton samples were collected during early summer 2022 from eight locations that were chosen to cover different ecological entities selected along Eastern Alexandria coast which were affected by natural and/or anthropic disturbances. These sites are Eastern Harbour (EH), Sidi Gaber (SG), Gleem (G), Abu Qir (AQ), Boughaz El Maadiya (M), Baltim (B), Ras El Bar (RB), and Port Said (PS) (Fig. 1 and Table 1). Stations EH and PS are commercial harbours which subject to different anthropogenic activities like sewage water and shipping, as well as stations AQ and B, that are affected by a large amount of untreated sewage, agricultural, and industrial, and wastewater, in addition to the existing trade ships in RB station. Stations G and SG are represented unpolluted areas since they are influenced by the water current that flows from the west to the east. El Maaddiya channel, which is approximately 100 m long, 20 m wide, and 3 m deep, is where water from Abu Qir Bay and Idku Lake is exchanged. This area is subjected to irregular changes as a result of the continuous mixing of Mediterranean Sea water with Lake Edku’s brackish water, which creates unstable ecological features (Gharib & Dorgham, 2000). In addition, it is received drainage water from Edku Lake which contains undefined levels of agricultural, industrial, and urban, chemicals from the Beheira Governorate and beyond.

**Table 1:** Location and characteristics of the sampling stations

	**Main characteristic**	**Latitude N**	**Longitude E**
Eastern Harbour	Shallow semi-enclosed basin, subjected to different anthropogenic effects from human activities including fishing, yacht sport, land-based effluents, and boat building	31.2081°	31° 12′ 29″
Sidi Gaber	It is unpolluted areas and affected by the water current from the west to the east	31.2234°	29.9368°
Gleem	Subjected to pollutants from the local resident	31.241478°	29.95749°
Abu Qir	Shallow semi-circular basin, affecting by Anthropogenic impact from shipping, commercial fishing, swimming, and recreational boating	31.311424°	30.06033°
El-Maadiya	It affected by a huge amount of untreated sewage and industrial waste waters. Swimming activities	31.10°	30.8°
Baltim		31.376112°	31.152°
Ras El bar	Anthropogenic impact from the local residents and the existing trade ships. Swimming activities	31.506048°	31.82778
Port Said	Commercial harbour receiving anthropogenic activity	31.26531°	32.3019

### Physicochemical parameters of the seawater

Surface measurements of water temperature, salinity, dissolved oxygen, and pH were operated using the water-checked physical parameter device (HANA, Model HI 9828). The dissolved inorganic nutrients (NO_2_, NO_3_, NH_4_, PO_4_, and SiO_4_) were analyzed following the methods of Parsons et al. (1984), and the chlorophyll *a* (Chl *a*) was estimated according to the Parsons (2013) method.

### Phytoplankton collection and identification

Surface water samples were obtained using a fine net (20-µm mesh) and trawled vertically (0.5 m/s) then filtered water was determined using a flowmeter (HYDRO-BIOS, Kiel, Germany). The collected samples were preserved in a neutralised formalin (4%), and a few drops of Lugol’s acid solution then stored in the dark before being transported to the Taxonomy and Biodiversity of Aquatic Biota laboratory, Alexandria, Egypt, for taxonomical examination and counting. The phytoplankton analysis was performed using an inverted microscope (Optika 100) at × 400 magnification after sedimentation (Utermöhl, 1958). The taxonomical identification of phytoplankton was done according to Krammer and Lange-Berlatot (1991) and Canter-Lund and Lund (1996) and then confirmed with the Algae Base website (Guiry & Guiry, 2022).

### Estimation of the water quality using different indices

There are different assays are used to estimate the water quality, including the phytoplankton abundance (Kitsiou & Karydis, 2001), chlorophyll *a* content index (Karydis, 1999), and modified by Simboura et al. (2005). Also, Eutrophication Index (E.I.) is a multi-metric (combination of nutrients and Chl *a*) tool for assessing trophic state (Primpas et al., 2010). The following formula is used to determine the eutrophication index:

$$\begin{aligned}\mathbf{E.I}=&\ 0.279\times \left[{PO}_{4}\right]+0.261\times \left[{NO}_{3}\right]+0.296\\&\times \left[{NO}_{2}\right] \ +0.275\times \left[{NH}_{3}\right]+0.214\times [Chl \;a]\end{aligned}$$

where E.I. < 0.04 (high ecological water quality), E.I. = 0.04–0.38 (good), E.I. = 0.38–0.85 (moderate), E.I. = 0.85–1.51 (poor), and E.I. > 1.51 (bad).

The species diversity (*H′*) (Shannon, 1997), species richness (*d*) (Margalef, 1978), and species evenness (*J*) (Pielou, 1975).

$$\mathbf{{H}^{\prime}}=3.3219\times [logN-\left(\frac{1}{N}\right)\Sigma ni\; logni]$$

where *N* is the total number of individuals of all species and *ni* is the number of individuals of a species.

$$\boldsymbol{d}=S-1/LnN$$

where *S* is the total number of species and *N* is the total number of individuals in the sample.

$$\boldsymbol{J}=H/S$$

where *H* is the Shannon index and *S* is the number of species.

The Palmer pollution index is used to detect the level of pollution in the environment based on the algal taxa found (Palmer, 1969).

### Similarity index

The similarity degree between phytoplankton species of the studied areas was calculated as a statistical parameter using Sorenson’s equation (Sorenson, 1948), which depends on the presence or absence of different species:

$$\mathrm{ISs}=\frac{2C\times 100}{A+B}$$

where

ISs: similarity quotient

C: species number common in both sites

A: species number in the first site

B: number of species in the second site

### Biochemical compositions of phytoplankton community

Plastic bottles were used to collect a known volume of seawater sample from the selected sites, which was subsequently sieved and filtered to separate macrozooplankton using a zooplankton net (100-µm mesh size). The samples were moved to the laboratory in ice tanks and re-filtered on Whatman GF/F (0.7-µm pore diameter) fibre circles to measure the biochemical characteristics of the separated phytoplankton. Total protein content (PRO) was determined by the Biuret method (David & Hazel, 1993). Total carbohydrate content (CHO) is conducted using the method established by Dubois et al. (1956). Total lipid content (LIP) is detected by following Bligh and Dyer (1959).

### Statistical analysis

Cluster analysis was performed using Primer 6.1.9 software (Primer-E Ltd.) to generate dendrograms (group average method), based on the Jaccard and Bray–Curtis distance matrixes among samples. One-way ANOVA test was used for determination of differences between stations in relation to various physciochemical properties of waters and phytoplankton biomass using SPSS software 30, 2020. Correlation coefficient (r) was estimated using the Microsoft Excel 2018 to evaluate the relation between the physicochemical parameters and phytoplankton abundance, groups, and their biochemical composition (*n* = 21) in the selected study areas.

## Results and discussion

### Physicochemical parameter

Spatial variations in water quality parameters along the Eastern Alexandria coast during the summer 2022 are tabulated in Table 2. Significant differences were observed in physcial and nutrient values between the studied stations (one-way ANOVA, *p* < 0.05). Temperature is characterized by a slightly different between stations; it was a minimum in B station (17 °C), and a peak of 21 °C is recorded in EH. A slightly alkaline range in pH is measured at the eight studied sites, and it varied between 7.94 at B station and 8.16 at RB and EH stations. Salinity is the primary physical parameter associated with plankton diversity and reflects the level of contamination in the aquatic environment (Zyadah et al., 2004). It displayed high oscillations with a maximum of 41.20 PSU in AQ and PS stations which is more than that of the Mediterranean water (38.50 PSU) (Copin-Montégut & Bégovic, 2002). Since, there are many natural factors like prolonged periods of heavy rain and terrestial runoff, as well as artificial sources like power plant discharges, can change the water salinity (Sew & Todd, 2020). A minimum pH value is recorded at B station 22.47 PSU reflecting the presence of fresh water source. Generally, the salinity values clearly reflect the effect of water discharge input. Dissolved oxygen is one of the most essential indicators in determining the degree of water pollution caused by organic contaminants that affect organisms’ life in a water body through oxygen reduction or depletion. Dissolved oxygen concentrations ranged from 4.5 mg L^−1^ at PS to 8 mg L^−1^ at EH.

**Table 2:** The average of physiochemical parameters of the selected areas

	**T (°C)**	**pH**	**Salinity (PSU)**	**Do (mg L**^**−1**^**)**	**PO**_**4**_**-P (Μmol L**^**−1**^**)**	**SiO**_**4**_**-Si (Μmol L**^**−1**^**)**	**NO**_**2**_**-N (Μmol L**^**−1**^**)**	**No**_**3**_**-N (Μmol L**^**−1**^**)**	**NH**_**4**_**-N (Μmol L**^**−1**^**)**
**Eastern Harbour**	21.00 ± 0.5^a^	8.16 ± 0.1^a^	38.24 ± 1.1^a^	8 ± 0.34^a^	0.672 ± 0.11^c^	3.192 ± 1.01^c^	0.8 ± 0.07^c^	11.2 ± 0.02^b^	7.63 ± 0.35^a^
**Sidi Gaber**	19.50 ± 1.0^b^	8.09 ± 0.12^a^	39.5 ± 0.45^a^	6.7 ± 1.2^a^	0.864 ± 0.15^c^	3.192 ± 0.98^c^	0.95 ± 0.08^c^	3.23 ± 0.12^d^	5.12 ± 0.54^b^
**Gleem**	20.50 ± 1.5^a^	8.14 ± 0.15^a^	36.3 ± 1.23^b^	7 ± 0.3^a^	0.816 ± 0.21^c^	6.91 ± 0.21^b^	0.98 ± 0.03^c^	18.78 ± 0.21^a^	3.55 ± 0.26^c^
**Abu Qir**	20.00 ± 0.5^a^	8.11 ± 0.11^a^	41.2 ± 0.5^a^	6.5 ± 0.21^a^	0.24 ± 0.13^d^	1.60 ± 0.31	2.65 ± 0.02^b^	16.19 ± 0.02^a^	8.2 ± 1.02^a^
**El-Maadiya**	19.00 ± 0.5^b^	7.96 ± 0.13^b^	32.11 ± 2.23^c^	5.8 ± 0.31^b^	1.728 ± 0.32^b^	2.51 ± 1.76^c^	5.55 ± 1.07^a^	12.14 ± 1.21^b^	5.04 ± 0.0^b^
**Baltim**	17.00 ± 1.0^c^	7.94 ± 0.12^b^	22.47 ± 2.54^d^	4.8 ± 0.32^c^	0.72 ± 0.12^c^	1.23 ± 1.67^d^	3.7 ± 0.25^b^	8.62 ± 1.09^c^	8.12 ± 0.87^a^
**Ras El Bar**	16.00 ± 1.0^c^	8.16 ± 0.12^a^	33.77 ± 1.32^c^	5.5 ± 0.23^b^	1.536 ± 0.23^b^	5.62 ± 1.32^b^	2.33 ± 0.22^b^	11.38 ± 1.32^b^	6.21 ± 0.02^b^
**Port Said**	18.50 ± 1.5^b^	8.0 ± 0.1^a^	41.20 ± 0.54^a^	4.5 ± 0.15^c^	5.616 ± 1.55^a^	10.72 ± 1.45^a^	2.63 ± 0.62^b^	4.86 ± 0.23^d^	5.85 ± 0.45^b^

Means with the same superscript letter in the same column were insignificantly different (*P < *0.05)

Means with the different superscript letter in the same column were significantly different (*P < *0.05)

Generally, seawater is characterized by high levels of nutrients, indicating influences of discharged water arrival. The total nitrogen explains the NO_2_, NO_3_, and NH_4_ concentrations. Nitrate is the highest component of inorganic nitrogen compounds, its concentration was varied from the lowest in AQ (0.19 µM) to the highest in G station (18.78 µM). The nitrite concentration ranged between 0.15 and 5.55 µM in AQ and M stations, respectively. Furthermore, concentrations differed dramatically between sites, ranging from 0.04 to 8.12 µM in M and B stations, respectively. The levels of NO_3_ (4 µM) and NH_4_ (2 µM) in most stations are the criteria of eutrophication (Oczkowski & Nioxn, 2008). Phosphate concentration fluctuated from 0.24 to 5.62 µM in AQ and PS stations, respectively. According to Stirn (1988) who reported that the soluble phosphorus in usually present in high quantity in the polluted water, therefore PS, m, and RB are considered a high polluted area compering to other selected stations. The N:P ratios in stations (EH, G, AQ, and B) were higher than the Redfield ratio (16:1), revealing a high nitrogen budget, meaning phosphorus was exerting a limiting effect in these stations. The SiO_4_ concentrations showed high oscillations between different sites with a peak in PS (10.72 µM) and lower concentration in AQ (1.60 µM). This wide range was related to the abundance of Bacillariophyceae (diatoms) (*r* = 0.61) (Table 2S). Spatial variation of Chl* a* (mean 1.22 µg L^−1^) experienced a wide range of variability at *p* < 0.05. Two distinct peaks (> 1 µg L^−1^) were measured in B and RB, accompanied by rapid phytoplankton proliferation (*r* = 0.74). However, an increase in both Chl *a* occurs in response to a rise in the phosphate and nitrate content of saltwater (Nassar & Hamed, 2003). This is consistent with the current findings, there was a significant positive association between Chl-*a* and phosphate (*r* = 0.719) and nitrate (*r* = 0.616) at *p* ≤ 0.05. In general, the regional variations of physicochemical properties of water may be attributed to wastewater discharged, such as drainage and fishing. Especially, the high concentrations of the nutrients (nitrate and phosphate) may be attributed to the impact of anthropogenic activities in cetain stations like fishing, swimming, wastes of agricultural, industrial, and sewage wastes. The overall average values of physicochemical parameters in the current study are more or less similar to that observed by previous researchers in Alexandria coast (Labib et al., 2023; Alprol et al., 2021).

### The structure and composition of the phytoplankton community

A total of 126 algal species were identified in the studied stations. Generally, the recorded species could be considered temperate water forms, eurythermal, are being reported previously in the Mediterranean Sea. Diatoms had the highest richness index (35 genera, 87 taxa) and abundance (91.06% of total abundance), followed by Dinoflagellates (Dinophyta) (14 genera, 30 taxa) comprised abundance (1.81%). Cyanophyceae were characterized by 4 species. Both Chlorophyceae and Silicoflagellata were represented by two species, and Euglenophyceae had one species (Table 1S). A comparison with previous studies revealed a more or less similar with Alprol et al. (2021) and Dango et al. (2015). In general, diatom species were the dominant group in all stations except in B station due to they have high adaptation, to survival in different waters including extreme conditions (Odum, 1998) so that they can be used as a bioindicator for unpolluted waters (Al-Tamimi & Al-Jumaily, 2021). Also, the dominance of diatoms was controlled by Si (*r* = 0.613) and PO_4_ (*r* = 0.555) because PO_4_ and SiO_4_ are the main factors controling the growth of phytoplankton (Rollwagen-Bollens & Connelly, 2022). Maximum density of Cyanophytceae was observed in B station due to the overgrowth of freshwater alga “*Microcystis aeruginos*a (Kützing)” which represented the dominant specie (82%). The presence of cyanobacteria indicated a low salinity (*r* = –0.85). A similar observation was detected by Cui et al. (2020) who reported that a high ratio of salt in seawater is known to reduce cyanobacterial growth. Also, the growth of Euglenophceae species might be stimulated by inorganic nitrogen content (NO_2_ (*r* = 0.83) and NH_4_ (*r* = 0.76)) (Table 2S). This finding was in agreement with Touliaba et al. (2010) who reported cyanobacteria species were more frequent in polluted water.

### Distribution and abundance of the phytoplankton

The phytoplankton abundance and biodiversity were significantly spatially variable in the study areas (one-way ANOVA, *p* < 0.05). This variation depended on the interactions of physical and chemical parameters, which were in turn influenced by climatic and anthropogenic activity (Kim et al., 2020). The correlation results show positive values between physicochemical parameters and the abundance of phytoplankton reflecting their vital role in the spatial variations of phytoplankton biomass. In general, the abundance of phytoplankton community is significantly related diatoms (*r* = 0.996) and dinoflagellates (*r* = 0.989) while they are regarded as the primary element of phytoplankton in the maritime environment (Limates et al., 2016). Also, ammonium plays a vital role in phytoplankton abundance (*r* = 0.76) since NH_4_ is the preferred source of nitrogen for phytoplankton (Straskraba & Tundisi, 1999). In addition to physciochemical parameters of water, there are numerous other factors that can affect the spatial distribution of phytoplankton, such as the turbidity maximum location and magnitude, the relative importance of tidal amplitude and freshwater discharge volume, water column stratification, and zooplankton grazing rates (Harrison et al., 1991).

**Fig. 2:**
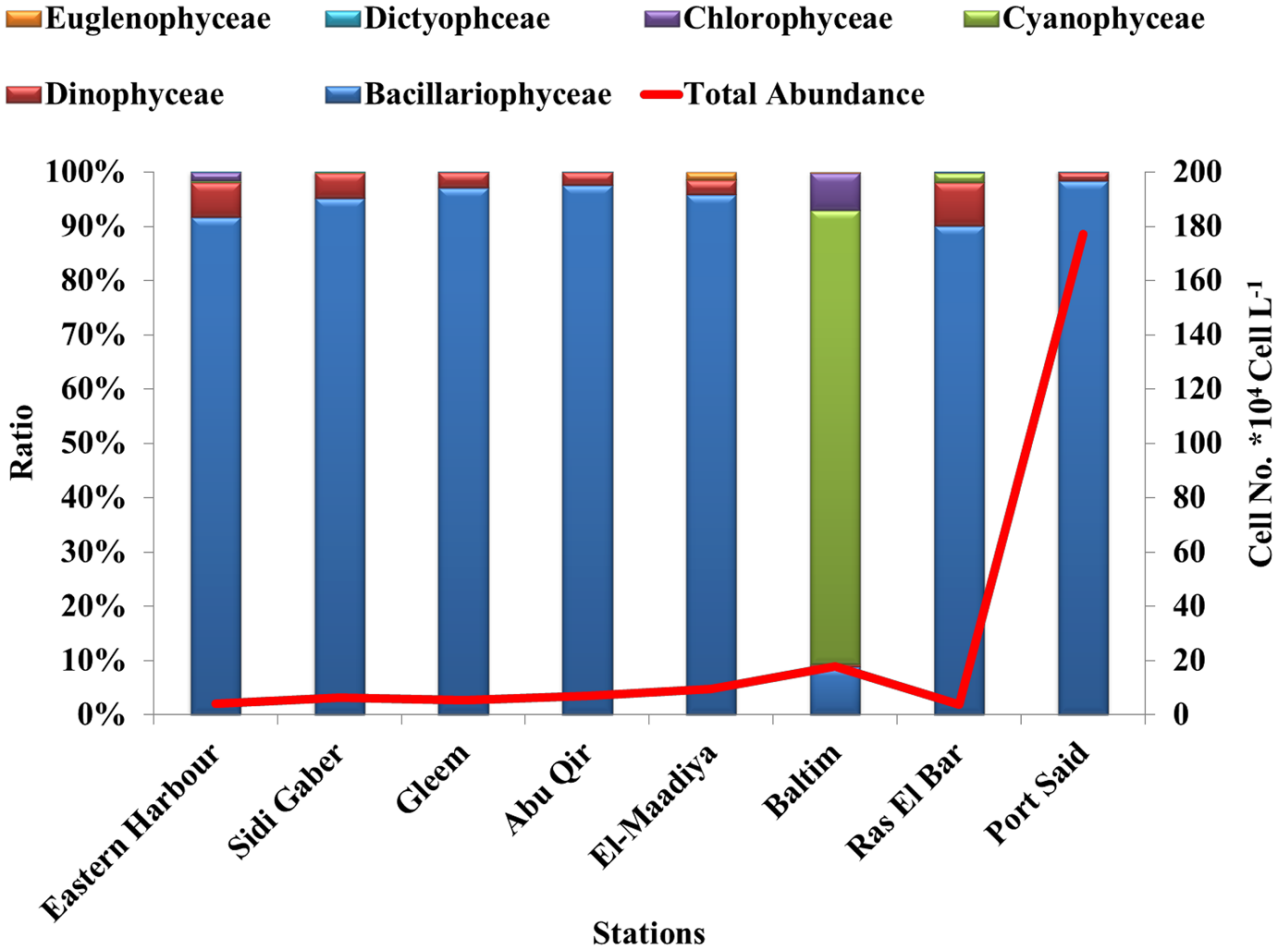
Spatial variation of phytoplankton community abundance (cell no. × 10^4^ cell L^−1^) and ratio %

The highest average of phytoplankton abundance occurs at PS coast (1.77 × 10^6^ cell L^−1^) followed by B (177 × 10^5^ cell L^−1^), M (963 × 10^4^ cell L^−1^), AQ (683 × 10^4^ cell L^−1^), SG (646 × 10^4^ cell L^−1^), and G (532 × 10^4^ cell L^−1^) as shown in Fig. 2. The lowest phytoplankton abundance was detected in EH (420 × 10^4^) and RB (375 × 10^4^ cell L^−1^). The highest abundance of phytoplankton at PS and B stations is related to the overgrowth of *Skeletonema costatum* (1.7 × 10^6^ cell L^−1^) and *Microcystis aeruginosa* (1.5 × 10^5^ cell L^−1^), respectively.

The range of the determined phytoplankton biomass is similar to that recorded previously at different Alexandria coasts (236.4 × 10^3^ cells L^−1^) (Zaghloul, 1994) and (680 × 10^3^ cells L^−1^) (Hussein, 2008). The species succession developed as *Skeletonema costatum* dominates in the EH (35.5%), *Actinoptychus splendens* in SG (20.7%),* Bacteriastrum hyalinum* in G (39.8%), *Melosira granulata* in AQ (28.3%), *Skeletonema costatum* in M, RB, and PS (31.9%, 52.7%, and 96%, respectively), and *Microcystis aeruginosa* in B station (82.5%).

According to the evaluation of phytoplankton cell density (Kitsiou & Karydis, 2001), all the selected stations along Eastern Mediterranean Sea water body were at a level of oligotrophic state (abundance < 3 × 10^5^) except PS (abundance > 10 × 10^5^) was eutrophic state.

Spatial fluctuation showed wide variation in dominant species. This include *Skeletonema costatum* (Greville) Cleve, which formed the bulk of the phytoplankton abundance at the most stations except at B stations. Previously, *S. costatum* has been identified as a blooming species that occasionally causes red tide in the Egyptian Mediterranean water, in Damietta branch mouth (Halim, 1960), Eastern Harbour (Ismail & Ibrahium, 2017), and Abu-Qir Bay (El-Sherif & Mikhail, 2003). Plus, *Skeletonema costatum* is a indicator species of eutrophication due to its high ability of pollution tolerance (Nassar & Hamed, 2003). Moreover, *Melosira granulata* (Ehrenberg) Ralfs, different *Chaetoceros* spp., *Pleurosigma rigidum* W. Smith, and *Pseudo-nitzschia pungens* (Grunow ex Cleve) Hasle were the dominant diatoms in the most stations. While *Microcystis aeruginosa* Kützing from Cyanophyta and *Chlorella vulgaris* Beijerinck from Chlorophyta appeared to be the often major component at B station. Also, at B and EH stations, only two species of green algae recorded and completely missed in other stations, namely *Chlorella vulgaris* Beijerinck and *Pediastrum clathratum* (Schröder) Lemmermann. Whereas, these green species could be used as a bioindicator or a natural biomarker of contaminations in aquatic environments (Okogwu & Ugwumba, 2013).

### Chlorophyll *a* concentration

The chlorophyll *a* concentration itself can be used as a guide in determining the trophic status of water. The highest Chl *a* content is detected in PS (3.24 µg L^−1^). Meanwhile, the lowest Chl *a* content (0.27 µg L^−1^) occurs at the EH beach.

Generally, the estimated Chl *a* in all the selected stations was lower than that detected by Alprol et al. (2021) 6.96 µg L^−1^ at eight stations along Southeastern Mediterranean Sea, Alexandria. Depending on the eutrophication scale based on Chl *a* concentrations proposed for the Greek Seas by Karydis (1999) and modified by Simboura et al*.* (2005) to comply with the five levels of ecological status defined by the Water Framework Directive has been also applied. Whereas, the eutrophication scale is divided into the categories according to Chl *a*: high-quality water system (< 0.1 µg L^−1^), oligotrophic (good) (0.1–0.4 µg L^−1^), mesotrophic (moderate) (0.4–0.6 µg L^−1^), poor (0.6–2.21 µg L^−1^), and bad (> 2.21 µg L^−1^). As shown in Table 3, the water quality fluctuated from good/moderate at the west (EH, SG, G, and AQ stations) to bad state at the east (M, B, RB, and PS stations).

**Table 3:** Eutrophication status of the studied areas depending on Chl *a* (µg L^−1^) and EI

Locations	Chl *a* content (µg L^−1^)	Karydis (1999) and Simboura et al. (2005)	Eutrophication Index (E.I.)	
Eastern Harbour	0.27	Good	4.67	Dystrophic/bad
Sidi Gaber	0.29	Good	2.83	Dystrophic/bad
Gleem	0.65	Moderate	6.53	Dystrophic/bad
Abu Qir	0.52	Moderate	1.43	Eutrophic/poor
El Maadiya	2.04	Poor	4.17	Dystrophic/bad
Baltim	2.44	Bad	8.13	Dystrophic/bad
Ras El bar	2.32	Bad	6.29	Dystrophic/bad
Port Said	3.24	Bad	5.92	Dystrophic/bad

A significant positive relationship was detected between Chl *a* and phytoplankton abundance (*r* = 0.74), especially diatoms (*r* = 0.69) and dinoflagellate (*r* = 0.65), which may explain the confined existence of these species to the studied areas. Also, Chl *a* was positively correlated with the NO_2_ (*r* = 0.616**)** and PO_4_ (*r* = 0.719) (*p* < 0.05) as detected by Lefebvre and Dezécache (2020).

### Eutrophication Index (E.I.)

The calculated E.I. values above the value 1.5, indicating generalized eutrophication, with the maximum value of 8.13 in B station and the lowest value at AQ (1.43), indicating all studied areas are a bad/poor trophic state (Table 3) which reflecting a significant ecological change of the marine system (Primpas et al., 2010; Simboura et al., 2016), and the reason for this may be to the different anthropogenic activities as mention in Table 1.

### Biodiversity indices

**Fig. 3:**
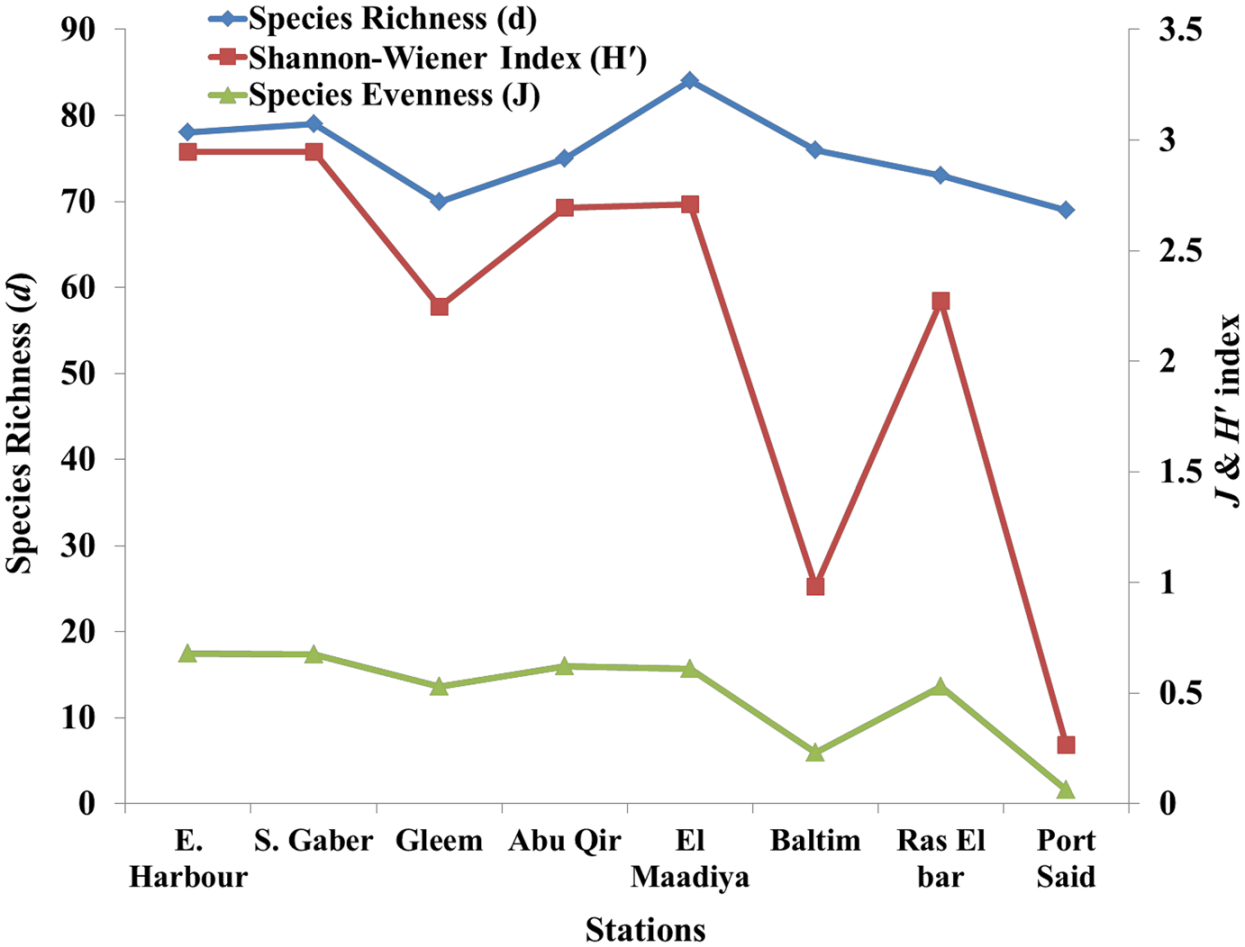
Richness, evenness, and the Shannon index of the phytoplankton groups along the Eastern of Alexandria coast

Indices of species diversity, evenness, and species richness were estimated as parameters to define the structure of the phytoplankton community (Fig. 3). There are many factors that can influence diversity like biotic factors such as predation or competition, as well as abiotic factors such as habitat harshness, heterogeneity, or size (Estrada et al., 2004). There are several numerical attempts have been made to express levels of oligotrophy and eutrophy based on phytoplankton species rather than nutritional water concentration. Based on Shannon classification, all stations can be sorted as moderate pollution (2–3 nats) except B (0.983 nats) and PS stations (0.266 nats) were a high pollution depending on Shannon et al. (1997) classification, whereas a low *H′* value indicated more pollution and high *H′* index value suggested a rich diversity. Also, the low value was met with low taxa number and the dominance of single or few species as previously observed the dominace of *M. aeruginos*a at B and *S. costatum* at PS station.

Moreover, the Pielou evenness index (*J*) was used to evaluate phytoplankton diversity at the selected stations. The phytoplankton J index ranged from 0.06 to 0.68 at PS and EH stations, with an average value of 0.49 which was consistent with the Shannon *H′* results. Whereas, the most selected stations were moderately polluted areas (0.5–0.6) except PS (0.06) and B (0.23) stations depending on the international standard classifications (Zheng et al., 2007).

As shown in Fig. 3, spatial variation in the phytoplankton richness (*d*) is detected along the Eastern Mediterranean, Egypt. The total number of species found on the examined sites showed a slight spatial difference. El Maadiya harbored 84 species, followed by 79 species was detected at SG. Close numbers of species have been recorded (73–78 spp.) at the RB, AQ, B, and EH stations, whereas only a small numaber (69–70 spp.) were observed at the PS and G stations, respectively. The biodiversity of the algal group varied depending on water properties. The richness of species is important factor in understanding the biodiversity and dynamics of communities. In our study, diatoms were important contributors to the species richness in all regions except B station. In general, the detected richness was lower than that observed in different Egypation Meditreanean coast by Alprol et al. (2021) (228 spp.) and El-Dahhar et al. (2021) (169 spp.).

### Palmer’s pollution index

Pollution indicator species are critical factors for determining biological water quality. The Palmer index proved to be an effective method for predicting pollution in surface water. The occurrence and distribution of palmer algal species are illustrated in Table 4. Since there are numerous species that can be used as bioindicators of ecosystem quality (Atıcı & Abel, 2016), eight pollution-tolerant algal taxa were detected in the studied areas. Based on the Palmer pollution index scores of phytoplankton species (Palmer, 1969), B and RB stations were probable organic pollution while the other stations showed no evidence of pollution since Palmer index scores < 10.

**Table 4:** Pollution index on the basis of palmer species occurrence and distribution in the studied water bodies

	**Eastern Harbour**	**Sidi Gaber**	**Gleem**	**Abu Qir**	**El-Maadiya**	**Baltim**	**Ras El Bar**	**Port Said**
*Cyclotella meneghinian*	0	0	0	0	0	0	3	0
*Melosira granulata*	0	1	0	1	1	0	0	1
*Navicula abrupta*	3	3	3	3	3	3	3	3
*Nitzschia acicularis*	0	3	0	3	0	0	0	0
*Synedra ulna*	2	0	0	0	0	2	2	2
*Oscillatoria nigroviridis*	0	0	0	0	0	5	0	0
*Chlorella vulgaris*	0	0	0	0	0	3	3	0
*Euglena granulata*	0	0	0	0	5	5	0	0
Total score	**5**	**7**	**3**	**7**	**9**	**18**	**11**	**6**

### Spatial similarity and site grouping

**Fig. 4:**
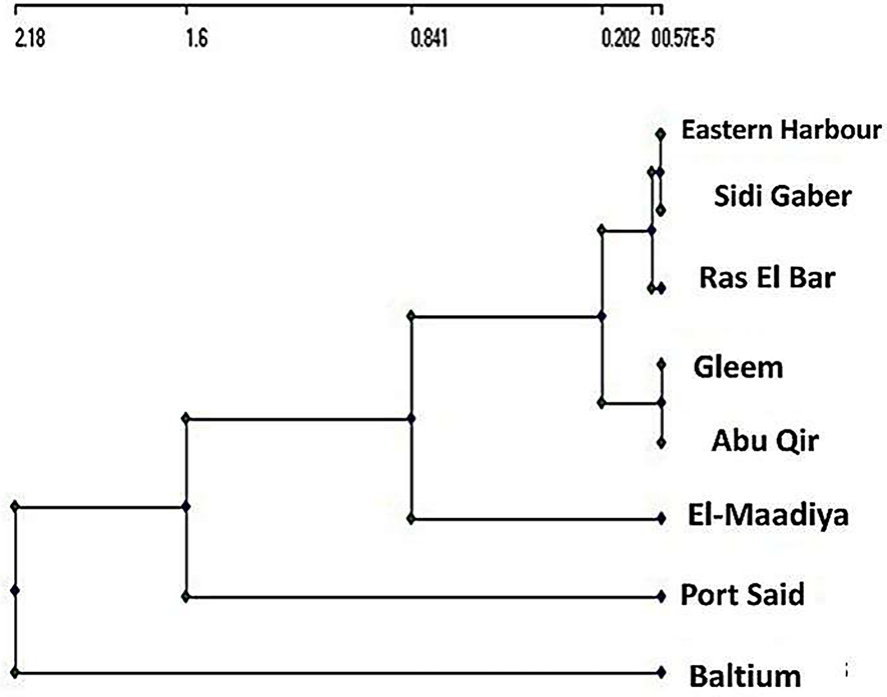
Clustering analysis for each monitoring site

To quantify the spatial variability within the phytoplankton population, we used the Bray–Curtis similarity index among stations. Cluster analysis is a statistical technique used to group similar variables into groups. With regard to the dendrogram, the sampling sites were divided into two statistically significant clusters. Cluster I included Baltim sampling site and all other selected areas in Cluster II. From examinations of the phytoplankton community composition (Fig. 4), since all areas had the same dominant group (diatom) except Baltim was characterized by the dominance of Cyanophyceae species.

Kulczynski coefficient similarity index was performed to find out the degree of similarity of the algal species compositions between the different stations (Table 5). The similarity index showed the stations B and M formed the highest similar pattern, comprising 66.20% similarity followed by the stations B and PS (60.44%), while the lowest similarity indices were detected between B and G stations (36.40%). This variation is related to algal species and abundance.

**Table 5:** Similarity index between stations

EH & SG	EH & G	EH & AQ	EH & M	EH & B	EH & RB	EH & PS
55.71	47.09	57.61	58.77	51.90	55.22	50.10
	SG & G	SG & AQ	SG & M	SG & B	SG & RB	SG & PS
	48.57	51.30	46.65	47.86	52.49	46.58
		G & AQ	G & M	G & B	G & RB	G & PS
		46.17	36.63	36.40	45.70	47.06
			AQ & M	M & B	E & RB	E & PS
			53.95	66.20	57.02	57.89
				AQ & B	B & RB	B & PS
				49.96	47.80	57.89
					AQ & RB	AQ & PS
					59.76	51.59
						RB & PS
						50.68

### Biochemical compositions of phytoplankton community

**Fig. 5:**
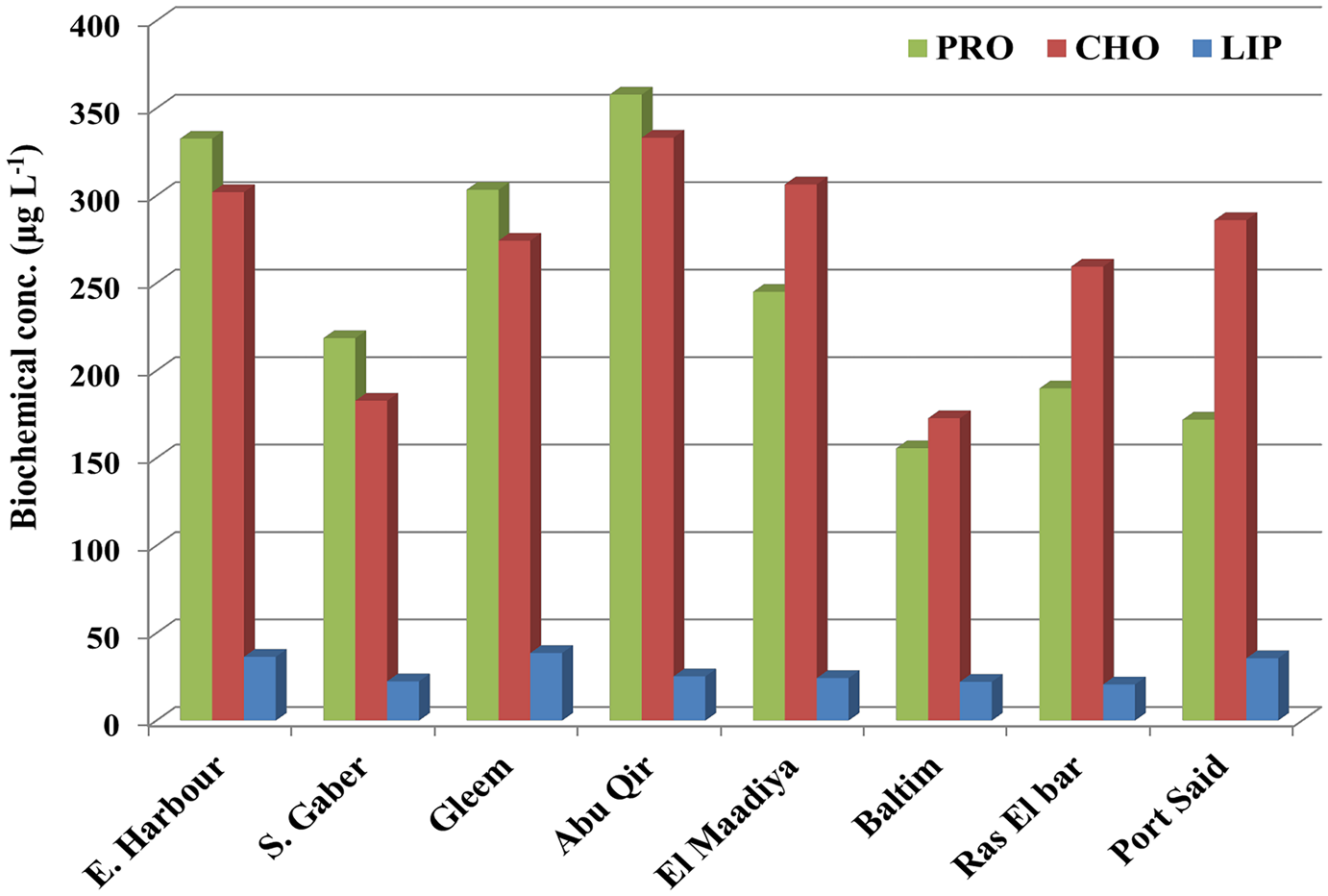
Biochemical contents (µg L^−1^) of the collected phytoplankton from different coast along Eastern Mediterranean sea Alexandria

Changes in cellular level biomarkers (PRT, CHO, and LIP contents) have the ability to clarify cellular biochemical responses that may accompany structural and functional modifications (primary productivity and nutrient absorption rates) following pollution exposure (Heraud et al., 2008). The biochemical patterns are used and employed as a biomethod to monitor changes in the algal physiological status as a result of climate change (Ahn et al., 2019; Ismail et al., 2022) and are utilized to determine the trophic condition (Kim et al., 2020). Globally, recent research has concentrated on how changes in water characteristics are related to variations in phytoplankton biochemical composition. However, there have been only a few studies in Egypt that have reported on such topics thus far. Biochemical compositions of phytoplankton in the studied stations showed significant spatial differences (Fig. 5) may be related to differences in phytoplankton classes and physicochemical parameters (Kim et al., 2022). Also, the algal growth phases have a major role in determining changes in their macromolecules contents (Fernández-Reiriz et al., 1989). Proteins represent the main biochemical content in the collected phytoplankton from EH, SG, G, and AQ stations followed by carbohydrates and lipids which generally reflect sufficient nitrogen conditions and physiologically healthy phytoplankton with high relative growth rates in relation to productive regions (Kim et al., 2022). On the other stations, the estimated CHO (av. 232.23 µg L^−1^) was the most abundant component in the phytoplankton biomass from M, B, RB, and PS stations, and PRO (av. 215.74 µg L^−1^) made the second contribution which suggests nitrogen starvation for phytoplankton growth in these stations (Danovaro et al., 2000). Also, Fathi et al. (2005) stated that increased CHO is caused by a drop in algal PRO in polluted areas.

The total phytoplankton lipid content (av. 28.13 µg L^−1^) represented the minor one (Fig. 5). Maximum LIP of 38.6, 36.4, and 35.6 µg L^−1^, respectively, were detected at G, EH, and PS, respectively. The detected macromolecules content was higher than the result stated by Ismail et al. (2022) at EH during 2020.

Proteins reached three major peaks; the highest in AQ (357.77 µg L^−1^) accompanied to the most abundant species *Chaetoceros decipience* and *Melosira granulata*; diatoms dominated the peak day (63 × 10^3^ cell L^−1^, Chl *a* 0.52 µg L^−1^). The second peak (303.33 µg L^−1^) in G station accompanied the initiation of the abundance of *Bacteriastrum hyalinum* (52 × 10^3^ cell L^−1^, Chl *a* 0.65 µg L^−1^). The protein contents of the phytoplankton community are significantly related total nitrogen content (*r* = 0.697) in water since proteins are nitrogenous compounds (Yun et al., 2015). The increase in protein is considered as an indicator that there is no nitrogen stress in phytoplankton metabolism (Ahn et al., 2019).

The proteins and carbohydrate ratio of the collected phytoplankton was decreased from west to east which suggests nitrogen limiting for the phytoplankton growth (Danovaro et al., 2000). This ratio has been used to characterize N-conditions and the physiological status of cells (Ismail et al., 2022; Kim et al., 2015). Under N-starvation conditions, both CHO and LIP were accumulated since they do not require N and serve as storage materials and structural components which they could be a reactional mechanism to survive (Hu, 2004).

Although there are limited data on the direct effects of water parameters on the macromolecules content of the phytoplankton community, but it is known that they are essential for algal growth. As a result of there is a decrease or/and an increase, there may be a reduction or/and stimulation the production of biochemical content (Boëchat & Giani., 2000). Based on the correlation analysis, the factor with the most significant impact on the biochemical content “CHO and LIP” of phytoplankton in this study was the temperature (Table 2S), whereas the water temperature is an important abiotic factor affecting the physiological activity of microbial communities and their growth (Zhao et al., 2020). The increasing temperature inhibits PRO synthesis (*r* =  − 0.67), consequently results in reduced algal growth rates (Schulte, 2015). Also, temperature had a negative impact on the LIP (*r* =  − 0.596) and CHO content (*r* =  − 0.641) as previously documented by Zhao et al. (2020). Moreover, salinity affects the biochemical content (PRO (*r* = 0.441) and CHO (*r* =  − 0.421)) of phytoplankton in the aquatic ecosystem. Similar observation was detected by Kumar and Saramma (2018) who demonstrated the increasing in PRO content and inihiation in CHO content with increasing the salinity. Also, PO_4_ showed a positive effect on CHO (*r* = 0.558) and PRO (*r* = 0.467) while phosphorus is a necessary nutrient that is involved in the CHO and PRO metabolic pathways of algae cells (Yaakob, 2021). The same trend was observed for PRO and total nitrogen (*r* = 0.691) and NO_3_ (*r* = 0.787), whereas nitrogen is the second most important nutrient for phytoplankton growth and the relative decrease in PRO was observed during N deficiency (Yaakob et al., 2021; Kim et al., 2022). Another factor seemed to affect the macromolecules content, namely DO concentration. Under low DO level, phytoplankton cells seemed to reduce their protein content (*r* = 0.708) as demonstrated by Boëchat and Giani (2000). Another factor that may have a significant impact on the biochemical components is the phytoplankton composition. Not only the algal species but also the growth phase (Boëchat & Giani, 2000). There is a relationship between diatoms and both CHO (*r* = 0.425) and LIP (*r* = 0.406), whereas the primary component of diatom cells is lipid, and the average lipid content in diatoms can reach 25% of dry weight (Levitan et al., 2014). Also, they produce a lot of extracellular polymeric compounds, such as glycoproteins and polysaccharides (Haynes et al., 2007).

## Conclusion

The quality of Egyptian Eastern Mediterranean Sea water is determined mainly depending on the phytoplankton community as a biological factor besides physical and chemical factors. This study is the first study on the relationship between the biochemical content of phytoplankton and the physicochemical parameters of water along different stations in the Alexandria coast. Generally, the most estimated index demonstrated the pollution rate increase toward the east. It can be concluded that the phytoplankton abundance, biodiversity, and biochemical content are suitable index for detecting the trophic state of the Alexandria coasts. Moreover, the estimation of phytoplankton is considered for its low cost and ease of data collection. Further studies on the spatial and temporal changes in the biochemical content of phytoplankton in relation to diverse environmental conditions are required to better understand the quantity and quality of the primary food source available to other biota.

### Supplementary Information

Below is the link to the electronic supplementary material.

Supplementary file1 (DOCX 61 KB)

## Data Availability

All data generated or analyzed during this study are included in this published article and its supplementary information file.
