# Supplementary material for: Trophic status determination of the Egyptian Eastern Mediterranean Sea based on phytoplankton diversity and their biochemical contents
Source: Environ Monit Assess. 2023 Aug 16;195(9):1040. doi: 10.1007/s10661-023-11690-z (PMC10427522; doi:10.1007/s10661-023-11690-z)
Supplement: Supplementary file 1 — Supplementary file1 (DOCX 61 KB) [file 10661_2023_11690_MOESM1_ESM.docx]

|  | **Table 1S Checklist of the identified phytoplankton species at the different studied stations.** | | | | | | | | |  |  |  |
| --- | --- | --- | --- | --- | --- | --- | --- | --- | --- | --- | --- | --- |
| **Algal spp.** | | **EH** | **SG** | **G** | **AQ** | **M** | **B** | **RB** | **PS** |  |  |  |
| **Bacillariophceae** | | | | | | | | | | | | |
| [***Actinastrum gracillimum*G.M.Smith**](https://www.algaebase.org/search/?genus=Actinastrum) | | | | - | - | - | - | - | + | - | - | |
| ***Actinoptychus splendens* (Shadbolt) Ralfs ex Pritchard** | | | | - | + | + | + | - | - | - | - | |
| ***Actinoptychus undulatus*(Kützing) Ralfs** | | | | + | + | + | + | + | + | + | + | |
| ***Amphipora alata* (Ehrenberg) Kützing** | | | | + | + | + | + | + | + | + | + | |
| ***Amphora grevilleana* var. prominens Grunow** | | | | + | + | + | + | + | + | + | + | |
| ***Amphora coffeiformis*(C.Agardh) Kützing** | | | | - | + | + | - | + | + | + | - | |
| ***Amphora hyalina* Kützing** | | | | + | + | + | + | + | + | + | + | |
| [***Amphora marina*W.Smith**](https://www.algaebase.org/search/?genus=Amphora) | | | | + | + | + | + | + | + | + | + | |
| ***Amphora lineolata* Ehrenberg** | | | | + | + | + | + | + | + | + | + | |
| [***Amphora ovalis*(Kützing) Kützing**](https://www.algaebase.org/search/?genus=Amphora) | | | | + | + | + | + | + | + | + | + | |
| ***Amphora turgida* W. Gregory** | | | | + | + | + | + | + | + | + | + | |
| [***Asterionella glacialis*Castracane**](https://www.algaebase.org/search/?genus=Asterionella) | | | | - | + | - | - | + | - | - | - | |
| ***Bacteriastrum delicatulum* Cleve** | | | | + | + | + | + | + | + | + | + | |
| [***Bacteriastrum hyalinum* Lauder**](https://www.algaebase.org/search/?genus=Bacteriastrum) | | | | - | - | + | + | - | - | - | - | |
| ***Biddulphia mobiliensis* (J.W.Bailey) Grunow** | | | | + | + | + | + | + | + | + | + | |
| ***Cerataulina bergonii* (H. Peragallo) F.Schütt** | | | | + | + | + | + | + | + | + | + | |
| ***Cerataulina pelagica* (Cleve) Hendey** | | | | + | + | + | + | + | + | + | + | |
| [***Chaetoceros affinis*Lauder**](https://www.algaebase.org/search/?genus=Chaetoceros) | | | | - | + | - | - | - | - | - | + | |
| [***Chaetoceros atlanticus*Cleve**](https://www.algaebase.org/search/?genus=Chaetoceros) | | | | + | + | + | + | + | + | + | + | |
| ***Chaetoceros constrictus* Gran** | | | | + | + | + | + | + | + | + | + | |
| [***Chaetoceros curvisetus*Cleve**](https://www.algaebase.org/search/?genus=Chaetoceros) | | | | + | + | - | - | + | - | - | - | |
| ***Chaetoceros decipiens*Cleve** | | | | + | - | - | + | + | - | - | - | |
| ***Chaetoceros densus (Cleve) Cleve*** | | | | - | + | - | - | - | - | - | - | |
| ***Chaetoceros didymus* Ehrenberg** | | | | + | + | + | + | + | + | + | + | |
| [***Chaetoceros tortissimus*Gran**](https://www.algaebase.org/search/?genus=Chaetoceros) | | | | - | - | - | - | + | - | - | - | |
| [***Cocconeis placentula*Ehrenberg**](https://www.algaebase.org/search/?genus=Cocconeis) | | | | - | - | - | - | + | - | - | + | |
| ***Coscinodiscus marginatus* Ehrenberg** | | | | + | + | + | + | + | + | + | + | |
| ***Coscinodiscus perforatus* Ehrenberg** | | | | + | + | + | + | + | + | + | + | |
| [***Coscinodiscus centralis*Ehrenberg**](https://www.algaebase.org/search/?genus=Coscinodiscus) | | | | + | + | - | - | - | + | - | - | |
| [***Coscinodiscus granii*L.F.Gough**](https://www.algaebase.org/search/?genus=Coscinodiscus) | | | | - | - | - | - | - | - | + | - | |
| [***Cyclotella meneghiniana*Kützing**](https://www.algaebase.org/search/?genus=Cyclotella) | | | | - | - | - | - | - | - | + | - | |
| ***Cyclotella nana*Hustedt** | | | | + | + | - | - | + | - | - | - | |
| [***Cyclotella striata*(Kützing) Grunow**](https://www.algaebase.org/search/?genus=Cyclotella) | | | | + | + | + | + | + | + | + | + | |
| ***Cylindrotheca closterium*(Ehrenberg) Reimann & J. C. Lewin** | | | | + | + | + | + | + | + | + | + | |
| **Table 1S**  **continued** | | | |  |  |  |  |  |  |  |  | |
| ***Cymbella aspera* (Ehrenberg) Cleve** | | | | + | + | + | + | + | + | + | + | |
| [***Dictyocha fibula*Ehrenberg**](https://www.algaebase.org/search/?genus=Dictyocha) | | | | + | - | - | - | - | - | - | - | |
| [***Diploneis smithii*(Brébisson) Cleve**](https://www.algaebase.org/search/?genus=Diploneis) | | | | - | - | + | - | - | - | - | - | |
| ***Diploneis bombus* (Ehrenberg) Ehrenberg** | | | | + | + | + | + | + | + | + | + | |
| [***Grammatophora oceanica*Ehrenberg**](https://www.algaebase.org/search/?genus=Grammatophora) | | | | + | - | + | - | - | - | - | - | |
| [***Gyrosigma acuminatum*(Kützing) Rabenhorst**](https://www.algaebase.org/search/?genus=Gyrosigma) | | | | + | - | - | + | - | - | - | - | |
| [***Gyrosigma balticum*(Ehrenberg) Rabenhorst**](https://www.algaebase.org/search/?genus=Gyrosigma) | | | | - | - | - | - | - | + | - | - | |
| [***Hemiaulus hauckii*Grunow ex Van Heurck**](https://www.algaebase.org/search/?genus=Hemiaulus) | | | | - | - | - | - | + | - | - | - | |
| ***Fragilaria construens* (Ehrenberg) Grunow** | | | | + | + | + | + | + | + | + | + | |
| ***Fragilaria pectinalis* (O.F.Müller) Lyngbye** | | | | + | + | + | + | + | + | + | + | |
| ***Grammatophora angulosa* Ehrenberg** | | | | + | + | + | + | + | + | + | + | |
| ***Grammatophora marina* (Lyngbye) Kützing** | | | | + | + | + | + | + | + | + | + | |
| ***Grammatophora oceanica* Ehrenberg** | | | | + | + | + | + | + | + | + | + | |
| ***Guinardia flaccida* (Castracane) H.Peragallo** | | | | + | + | + | + | + | + | + | + | |
| ***Gyrosigma acuminatum* (Kützing) Rabenhorst** | | | | + | + | + | + | + | + | + | + | |
| ***Gyrosigma balticum*(Ehrenberg) Rabenhorst** | | | | + | + | + | + | + | + | + | + | |
| ***Hemiaulus hauckii* Grunow ex Van Heurck** | | | | + | + | + | + | + | + | + | + | |
| [***Lauderia borealis*Gran**](https://www.algaebase.org/search/?genus=Lauderia) | | | | - | - | - | + | - | - | - | - | |
| ***Leptocylindrus danicus* Cleve** | | | | + | + | + | + | + | + | + | + | |
| [***Licmophora abbreviata* C.Agardh**](https://www.algaebase.org/search/?genus=Licmophora) | | | | - | - | - | + | - | - | - | - | |
| [***Licmophora gracilis*(Ehrenberg) Grunow**](https://www.algaebase.org/search/?genus=Licmophora) | | | | - | - | - | + | + | - | - | - | |
| [***Licmophora paradoxa*(Lyngbye) C.Agardh**](https://www.algaebase.org/search/?genus=Licmophora) | | | | - | - | + | - | - | - | - | - | |
| [***Melosira granulata*(Ehrenberg) Ralfs**](https://www.algaebase.org/search/?genus=Melosira) | | | | - | + | - | + | + | - | - | + | |
| [***Melosira varians*C.Agardh**](https://www.algaebase.org/search/?genus=Melosira) | | | | - | - | + | - | - | - | - | - | |
| ***Navicula abrupta* (W.Gregory) Donkin** | | | | + | + | + | + | + | + | + | + | |
| ***Navicula dicephala var. neglecta Hustedt*** | | | | + | + | + | + | + | + | + | + | |
| ***Navicula humerosa* Brébisson ex W.Smith** | | | | + | + | + | + | + | + | + | + | |
| [***Navicula distans*(W.Smith) Brébisson**](https://www.algaebase.org/search/?genus=Navicula) | | | | - | - | - | - | - | + | + | - | |
| [***Navicula lyra*Ehrenberg**](https://www.algaebase.org/search/?genus=Navicula) | | | | - | + | - | - | - | - | - | - | |
| [***Nitzschia acicularis*(Kützing) W.Smith**](https://www.algaebase.org/search/?genus=Nitzschia) | | | | - | + | - | + | - | - | - | - | |
| ***Nitzschia delicatissima* Cleve** | | | | + | + | + | + | + | + | + | + | |
| ***Nitzschia longissima (Brébisson) Ralfs*** | | | | + | + | + | + | + | + | + | + | |
| [***Nitzschia sigma*(Kützing) W.Smith**](https://www.algaebase.org/search/?genus=Nitzschia) | | | | + | - | - | - | - | - | - | - | |
| **Table 1S**  **continued** | | | |  |  |  |  |  |  |  |  | |
| [***Odontella aurita*(Lyngbye) C.Agardh**](https://www.algaebase.org/search/?genus=Odontella) | | | | + | - | - | + | - | - | - | - | |
| ***Odontella obtusa Kützing*** | | | | - | + | + | - | + | + | - | - | |
| [***Plagiotropis lepidoptera* (W.Gregory) Kuntze**](https://www.algaebase.org/search/?genus=Plagiotropis) | | | | - | - | - | + | - | - | - | - | |
| [***Pleurosigma decorum*W.Smith**](https://www.algaebase.org/search/?genus=Pleurosigma) | | | | - | + | - | - | - | - | - | - | |
| [***Pleurosigma rigidum*W.Smith**](https://www.algaebase.org/search/?genus=Pleurosigma) | | | | - | - | - | + | + | - | - | - | |
| [***Pseudo-nitzschia pungens*(Grunow ex Cleve) Hasle**](https://www.algaebase.org/search/?genus=Pseudo-nitzschia) | | | | - | + | - | - | - | - | - | + | |
| ***Pseudonitzschia longissima* (Brébisson) Ralfs** | | | | + | + | + | + | + | + | + | + | |
| ***Pseudonitzschia lorenziana* Grunow** | | | | + | + | + | + | + | + | + | + | |
| ***Pseudonitzschia microcephala* Grunow** | | | | + | + | + | + | + | + | + | + | |
| ***Rhizosolenia alata* f. gracillima (Cleve) Grunow** | | | | + | + | + | + | + | + | + | + | |
| ***Rhizosolenia calcar-avis Schultze*** | | | | + | + | + | + | + | + | + | + | |
| [***Rhizosolenia fragilissima*Bergon**](https://www.algaebase.org/search/?genus=Rhizosolenia) | | | | - | - | - | - | + | - | - | - | |
| [***Rhizosolenia hebetata* J.W.Bailey**](https://www.algaebase.org/search/?genus=Rhizosolenia) | | | | - | - | - | - | - | - | - | + | |
| ***Schroederella delicatula* (Peragallo) Pavillard** | | | | + | + | + | - | + | - | + | - | |
| ***Skeletonema costatum* (Greville) Clev** | | | | + | + | + | - | + | - | + | + | |
| ***Striatella unipunctata*(Lyngbye)C. Agardh** | | | | + | + | + | + | + | + | - | + | |
| [***Surirella minuta*Brébisson ex Kützing, nom. illeg.**](https://www.algaebase.org/search/?genus=Surirella) | | | | + | + | + | + | + | + | + | + | |
| [***Synedra ulna*(Nitzsch) Ehrenberg**](https://www.algaebase.org/search/?genus=Synedra) | | | | + | - | - | - | - | + | + | + | |
| [***Thalassionema nitzschioides* (Grunow) Mereschkowsky**](https://www.algaebase.org/search/?genus=Thalassionema) | | | | - | + | - | - | + | + | - | + | |
| [***Thalassiothrix frauenfeldii*(Grunow) Grunow**](https://www.algaebase.org/search/?genus=Thalassiothrix) | | | | + | + | + | + | + | + | + | + | |
| ***Alexandrium catenella*** | | | | + | + | + | + | + | + | + | + | |
|  | **Dinophyta** | | | | | | | | | | | |
| [***Alexandrium minutum*Halim**](https://www.algaebase.org/search/?genus=Alexandrium) | | | | - | - | - | + | - | - | - | - | |
| ***Alexandrium ostenfeldii*(Paulsen) Balech & Tangen** | | | | + | + | + | + | + | + | + | + | |
| [***Cerataulina pelagica*(Cleve) Hendey**](https://www.algaebase.org/search/?genus=Cerataulina) | | | | - | - | + | - | + | + | - | + | |
| ***Ceratium breve*(Ostenfeld & Schmidt) Schröder** | | | | - | - | - | + | - | - | - | - | |
| [***Ceratium extensum*(Gourret) A.Cleve**](https://www.algaebase.org/search/?genus=Ceratium) | | | | - | - | - | + | + | - | - | - | |
| ***Ceratium furca*(Ehrenberg) Claparède & Lachmann** | | | | + | - | - | - | - | - | - | - | |
| ***Ceratium fusus* (Ehrenberg) Dujardin** | | | | + | + | + | + | + | + | + | + | |
| ***Ceratium tripos* (O.F.Müller) Nitzsch** | | | | + | + | + | + | + | + | + | + | |
| ***Dissodinium pseudolunula*** | | | | + | + | + | + | + | + | + | + | |
| ***Dinophysis caudata* W.S. Kent** | | | | + | + | + | + | + | + | + | + | |
| ***Diploneis bombus* (Ehr.) Clevé.** | | | | + | + | + | + | + | + | + | + | |
| **Table 1S**  **continued** | | | |  |  |  |  |  |  |  |  | |
| ***Diploneis notabilis*(Greville) Cleve** | | | | + | + | + | + | + | + | + | + | |
| ***Diplopsalis lenticula* Bergh** | | | | + | + | + | + | + | + | + | + | |
| ***Gonyaulax spinifera* (Claparède & Lachmann) Diesing** | | | | - | - | - | - | - | - | + | - | |
| [***Oxyphysis oxytoxoides* Kofoid**](https://www.algaebase.org/search/?genus=Oxyphysis) | | | | + | - | - | - | - | - | - | - | |
| ***Podolampas spinifera Okamura*** | | | | + | + | + | + | + | + | + | + | |
| ***Prorocentrum cordatum*(Ostenfeld) J.D.Dodg** | | | | + | + | + | + | + | + | + | + | |
| ***Prorocentrum gracile* F.Schütt** | | | | - | - | - | + | - | - | - | - | |
| [***Prorocentrum micans*Ehrenberg**](https://www.algaebase.org/search/?genus=Prorocentrum) | | | | - | - | - | + | - | - | - | + | |
| ***Prorocentrum oporum* (Schiller) Abe** | | | | + | + | + | + | + | + | + | + | |
| ***Prorocentrum sigmoides* Böhm** | | | | + | - | - | - | - | - | - | - | |
| [***Prorocentrum triestinum*J.Schiller**](https://www.algaebase.org/search/?genus=Prorocentrum) | | | | - | - | - | - | - | - | + | - | |
| [***Protoperidinium depressum*(Bailey) Balech**](https://www.algaebase.org/search/?genus=Protoperidinium) | | | | + | - | + | - | + | - | - | - | |
| ***Protoperidinium granii* (Ostenfeld) Balech** | | | | + | + | + | + | + | + | + | + | |
| [***Protoperidinium minutum*(Kofoid) Loeblich III**](https://www.algaebase.org/search/?genus=Protoperidinium) | | | | - | + | - | - | - | - | - | - | |
| ***Protoperidinium pellucidum* Bergh** | | | | + | + | + | + | + | + | + | + | |
| ***Protoperidinium steinii*(Jørgensen) Balech** | | | | - | - | - | - | + | - | - | - | |
| [***Pyrophacus horologium* F.Stein**](https://www.algaebase.org/search/?genus=Pyrophacus) | | | | - | - | - | - | + | - | - | - | |
| [***Scrippsiella trochoidea*(F.Stein) A.R.Loeblich**](https://www.algaebase.org/search/?genus=Scrippsiella) | | | | - | - | - | - | + | - | - | + | |
|  | | | |  |  | **Cyanophyta** |  |  |  |  |  | |
| ***Anabeana* sp.** | | | | + | + | - | - | + | + | + | - | |
| **Gomphosphaeria** **lacustris var. compacta Lemm .** | | | | - | - | - | - | - | + | + | - | |
| [***Microcystis aeruginos*a (Kützing) Kützing**](https://www.algaebase.org/search/?genus=Microcystis) | | | | - | - | - | - | - | + | + | - | |
| [***Oscillatoria nigroviridis* Thwaites ex Gomont**](https://www.algaebase.org/search/?genus=Oscillatoria) | | | | - | - | - | - | - | + | - | - | |
|  | **Chlorophyta** | | | | | | | | | | | |
| [***Chlorella vulgaris*Beijerinck**](https://www.algaebase.org/search/?genus=Chlorella) | | | | - | - | - | - | - | + | + | - | |
| [***Pediastrum clathratum*(Schröder) Lemmermann**](https://www.algaebase.org/search/?genus=Pediastrum) | | | | + | - | - | - | - | + | + | - | |
|  | **Silicoflagellata** | | | | | | | | | | | |
| ***Dictyocha fibula* Ehrenberg** | | | | + | + | + | + | + | + | + | + | |
| ***Dictyocha speculum* Ehrenberg** | | | | + | + | - | - | + | + | + | - | |
| **Euglenophyceae** | | | | | | | | | | | | |
| ***Euglena granulata*(G.A.Klebs) F.Schmitz** | | | | - | - | - | - | + | + | - | - | |

| **Table 2S** Pearson correlation matrix of the physico-chemical parameters and phytoplankton community and their biochemical content | | | | | | | | | | | | | | | | | | | | | |
| --- | --- | --- | --- | --- | --- | --- | --- | --- | --- | --- | --- | --- | --- | --- | --- | --- | --- | --- | --- | --- | --- |
|  | **T** | **Sal.** | **pH** | **Do** | **NO_3_** | **NO_2_** | **NH_4_** | **PO_4_** | **SiO_4_** | **T N** | **Chl *a*** | **Bacil.** | **Din.** | **Cyan** | **Chl** | **Silic.** | **Eug.** | **Abundance** | **LIP** | **CHO** | **PRO** |
| **T** | 1.00 |  |  |  |  |  |  |  |  |  |  |  |  |  |  |  |  |  |  |  |  |
| **Sal.** | 0.569 | 1.00 |  |  |  |  |  |  |  |  |  |  |  |  |  |  |  |  |  |  |  |
| **pH** | 0.331 | **0.538** | 1.00 |  |  |  |  |  |  |  |  |  |  |  |  |  |  |  |  |  |  |
| **Do** | 0.782 | 0.399 | 0.704 | 1.00 |  |  |  |  |  |  |  |  |  |  |  |  |  |  |  |  |  |
| **NO_3_** | 0.316 | -0.013 | 0.381 | 0.398 | 1.00 |  |  |  |  |  |  |  |  |  |  |  |  |  |  |  |  |
| **NO_2_** | -0.424 | *-0.509* | -0.782 | -0.604 | 0.006 | 1.00 |  |  |  |  |  |  |  |  |  |  |  |  |  |  |  |
| **NH_4_** | -0.167 | -0.196 | -0.090 | -0.081 | -0.086 | 0.122 | 1.00 |  |  |  |  |  |  |  |  |  |  |  |  |  |  |
| **PO_4_** | -0.235 | 0.268 | -0.362 | *-0.618* | *-0.477* | 0.182 | -0.220 | 1.00 |  |  |  |  |  |  |  |  |  |  |  |  |  |
| **SiO_4_** | 0.178 | **0.669** | -0.064 | -0.204 | -0.079 | 0.134 | *-0.523* | **0.624** | 1.00 |  |  |  |  |  |  |  |  |  |  |  |  |
| **T N** | 0.122 | -0.216 | 0.103 | 0.172 | 0.901 | 0.327 | 0.249 | -0.454 | -0.189 | 1.00 |  |  |  |  |  |  |  |  |  |  |  |
| **Chl *a*** | -0.331 | -0.319 | *-0.868* | *-0.797* | -0.366 | **0.616** | 0.032 | **0.719** | 0.245 | -0.153 | 1.00 |  |  |  |  |  |  |  |  |  |  |
| **Bacil.** | -0.086 | 0.379 | -0.318 | *-0.544* | *-0.452* | 0.059 | -0.105 | **0.963** | **0.613** | *-0.432* | **0.694** | 1.00 |  |  |  |  |  |  |  |  |  |
| **Din.** | -0.098 | 0.404 | -0.275 | *-0.522* | *-0.486* | 0.024 | -0.113 | **0.968** | **0.611** | *-0.476* | **0.657** | **0.997** | 1.00 |  |  |  |  |  |  |  |  |
| **Cyan** | *-0.458* | *-0.850* | *-0.581* | *-0.448* | -0.168 | 0.312 | **0.463** | -0.189 | *-0.693* | 0.070 | 0.418 | -0.168 | -0.204 | 1.00 |  |  |  |  |  |  |  |
| **Chl** | *-0.434* | *-0.846* | *-0.562* | -0.415 | -0.167 | 0.291 | **0.485** | -0.201 | *-0.721* | 0.071 | 0.403 | -0.177 | -0.212 | **0.999** | 1.00 |  |  |  |  |  |  |
| **Silic.** | *-0.652* | *-0.853* | -0.332 | -0.294 | -0.310 | 0.290 | 0.386 | -0.304 | *-0.770* | -0.090 | 0.089 | -0.416 | -0.405 | **0.756** | **0.762** | 1.00 |  |  |  |  |  |
| **Eug.** | -0.063 | -0.372 | *-0.597* | -0.180 | 0.076 | **0.834** | -0.209 | 0.016 | 0.173 | 0.247 | 0.356 | -0.145 | -0.164 | 0.025 | 0.016 | 0.164 | 1.00 |  |  |  |  |
| **Abundance** | -0.130 | 0.304 | -0.376 | *-0.592* | *-0.473* | 0.089 | -0.063 | **0.956** | **0.555** | *-0.431* | **0.742** | **0.996** | **0.989** | -0.076 | -0.086 | -0.350 | -0.144 | 1.00 |  |  |  |
| **LIP** | **-***0.596* | **0.429** | 0.270 | 0.364 | 0.271 | *-0.458* | -0.293 | 0.310 | 0.204 | 0.033 | 0.080 | **0.406** | 0.397 | -0.332 | -0.309 | *-0.618* | -0.273 | 0.380 | 1.00 |  |  |
| **CHO** | *-0.641* | *-0.421* | *-0.489* | *-0.675* | 0.180 | **0.558** | -0.250 | **0.544** | 0.188 | 0.253 | **0.708** | **0.425** | 0.392 | 0.239 | 0.219 | 0.058 | 0.357 | **0.452** | 0.167 | 1.00 |  |
| **PRO** | *-0.673* | **0.441** | **0.513** | **0.708** | **0.787** | -0.215 | 0.085 | ***0.467*** | 0.076 | **0.691** | *-0.513* | -0.345 | -0.365 | *-0.449* | *-0.432* | *-0.585* | -0.041 | -0.391 | 0.392 | -0.301 | 1.000 |

The bold value reflecting significant value at 0.05 level.

Whereas: TN (total nitrogen); Bacil. (Bacillariophyceae); Din. (Dinophyceae); Cyan. (Cyanophyceae); Chl. (Chlorophyta); Silic. (Silicoflagellata); Eug. (Euglenophyceae); LIP (Lipid); CHO (Carbohydrate); PRO (Protein).
